# Cervical Screening Systems in Eastern Europe and Central Asia: A Comparative Policy Evaluation

**DOI:** 10.3390/healthcare13222889

**Published:** 2025-11-13

**Authors:** Silvia Ussai, Teymur Seyidov, Nerea Blanqué Catalina, Tamar Khomasuridze

**Affiliations:** 1 UNFPA, Regional Office for Eastern Europe and Central Asia, Istanbul 34349, Turkey; 2 Alira Health, 08029 Barcelona, Spain

**Keywords:** cervical screening, Eastern Europe and Central Asia, United Nations Population Fund (UNFPA), health policy, Regional Alliance for Cervical Cancer Prevention

## Abstract

**Background/Objectives**: Cervical cancer remains a major cause of morbidity and mortality in Eastern Europe and Central Asia (EECA), where screening implementation remains fragmented. This study provides a comparative assessment of national cervical screening programs across the region, highlighting structural strengths and policy gaps. **Methods**: National self-reported data were collected through a standardized UNFPA questionnaire from 18 submissions representing 16 EECA countries and territories. Descriptive analyses compared organized and opportunistic approaches, and an illustrative Program Maturity Index (PMI) was constructed from eight structural and performance indicators aligned with WHO and European standards. **Results**: Fifteen submissions reported national screening guidelines and seventeen defined intervals, most commonly every three or five years. Organized systems achieved higher participation (median 57.2%) than opportunistic models (15%). Follow-up of screen-positive women was the weakest component, with rates ranging from below 2% to above 90%. The regional mean PMI was 0.73, reflecting intermediate-to-advanced maturity overall, but persistent gaps in monitoring and patient linkage to care. **Conclusions**: This study offers the first regional comparison of cervical screening systems in EECA. Despite policy progress, weak follow-up and incomplete data systems limit impact. Strengthening electronic registries, financing linkage to outcomes, and regional collaboration are essential to meet the WHO’s 90–70–90 elimination targets.

## 1. Introduction

Cervical cancer remains a major public health concern worldwide, with over 600,000 new cases and 340,000 deaths estimated annually, the majority of which occur in low- and middle-income countries [1]. The Eastern Europe and Central Asia (EECA) region carries a disproportionate burden, with age-standardized incidence and mortality rates significantly higher than in Western Europe in women of reproductive ages [2]. Despite the availability of effective screening and the potential for elimination as a public health problem, substantial disparities persist in program implementation, quality assurance, and population coverage [3].

Organized, population-based cervical screening programs have demonstrated superior effectiveness in reducing incidence and mortality compared to opportunistic approaches [4]. Key elements of successful programs include evidence-based national guidelines, defined target age groups, systematic invitation and recall mechanisms, and robust follow-up systems for screen-positive women [5]. The World Health Organization (WHO) and European Guidelines for Quality Assurance in Cervical Cancer Screening provide comprehensive frameworks for establishing and evaluating such programs [6,7].

In the EECA region, cervical screening systems are heterogeneous, ranging from fully organized national programs to opportunistic models with limited policy and infrastructure support [8]. Barriers to effective screening include inconsistent funding, lack of standardized invitation and follow-up protocols, and insufficient monitoring systems [9]. Previous comparative analyses have examined country-to-country variations in cervical cancer screening organization [10]; however, systematic regional comparisons for Eastern Europe and Central Asia (EECA) remain limited, despite the region’s diverse health systems and transition economies that strongly influence screening models and outcomes.

To accelerate progress towards the WHO Global Strategy for Cervical Cancer Elimination, which targets 70% screening coverage of women by 35 and again by 45 years of age [11], a detailed assessment of the maturity of national programs is urgently needed.

This study evaluates cervical screening programs across 16 EECA countries, comparing organized and opportunistic models and assessing key policy and performance indicators. In addition to analyzing participation rates, we developed a composite Program Maturity Index (PMI) to quantify the developmental stage of each program and identify regional trends to inform evidence-based policy recommendations.

The primary aim of this study was to provide a descriptive assessment of cervical screening programs across Eastern Europe and Central Asia (EECA). Specifically, the study sought to:

- Compare participation rates, where available, between organized, population-based programs and opportunistic models;
- Assess the presence and completeness of critical program elements, including national guidelines, defined target age groups, screening intervals, invitation systems, and follow-up mechanisms;
- Identify gaps and regional trends to inform policy recommendations aligned with WHO’s Global Strategy for Cervical Cancer Elimination [12].

The primary research question guiding this study was: How do structural and organizational features of national screening programs in EECA influence participation and follow-up outcomes?

In addition, an illustrative Program Maturity Index (PMI) was developed and applied in an exploratory manner to summarize the developmental stage of national programs and highlight regional variability, while acknowledging the limitations of available data.

## 2. Materials and Methods

This descriptive study utilized a standardized survey questionnaire developed by the UNFPA Regional Office to collect country-level data on cervical cancer in Eastern Europe and Central Asia (Supplementary Table S1). The aim was to compare policy frameworks, service delivery structures, and performance indicators of cervical screening programs across 16 countries and territories in Eastern Europe and Central Asia (Albania, Armenia, Republic of Belarus, Bosnia and Herzegovina, Georgia, Kazakhstan, Kyrgyzstan, Republic of Moldova, North Macedonia, Serbia, Tajikistan, Türkiye, Turkmenistan, Ukraine, and Uzbekistan, as well as Kosovo under UNSCR 1244). Bosnia and Herzegovina (BiH) were represented by three administrative entities (Federation of BiH, Republika Srpska, and Brčko District), resulting in 18 unique submissions. Data were collected at the national program level, rather than at the individual patient level, to assess policy frameworks, service delivery structures, and performance indicators. Countries included in the analysis represented a range of program types, from early-stage opportunistic approaches to fully organized, population-based screening systems. The population of interest consisted of women within each country’s defined target age group for cervical screening, as specified in national policies or practice guidelines. The questionnaire was developed by the UNFPA Regional Office through an iterative consultation with national SRH focal points and validated by the Regional Alliance experts. Responses were cross-checked by UNFPA country offices for internal consistency and accuracy to reduce reporting bias.

Countries were categorized as having either organized or opportunistic screening programs based on the structure of their national systems. Programs were classified as organized when they demonstrated population-based approaches with systematic invitation, centralized data collection, and defined follow-up protocols in line with European Guidelines for Quality Assurance in Cervical Cancer Screening, even when implementation was limited to pilot regions rather than nationwide. Countries relying on non-systematic, provider-driven approaches were classified as opportunistic.

Participation rate was defined as the percentage of invited women who underwent screening within the reporting period. Descriptive statistics (mean, median, range) were calculated separately for organized and opportunistic programs. Given the small number of opportunistic programs and variable data availability, non-parametric approaches were used for comparisons.

To complement the descriptive and comparative findings, we constructed an illustrative Program Maturity Index (PMI) based on eight structural and performance indicators aligned with WHO and European quality assurance guidance: program type (organized vs. opportunistic), availability of national guidelines, definition of screening interval, definition of target age group, existence of follow-up mechanisms for screen-positive women, state financing coverage, invitation systems, and participation rates. Each indicator was scored on a binary or scaled basis, with follow-up and participation scored proportionally according to reported quantitative values. The mean PMI score was then calculated for each submission to provide an overall measure of maturity. The PMI was applied exploratorily and for illustrative purposes only, intended to complement the descriptive findings and highlight regional variability rather than to provide a definitive ranking of program maturity.

### 2.1. Interpretation Thresholds

The PMI for each submission was calculated as the arithmetic mean of all eight indicator scores:

PMI = (Σx_i_)/8

where x_1_–x_8_ represent standardized indicator values.

Classification thresholds were derived by dividing the PMI scale (0.00–1.00) into five equal intervals to facilitate interpretation of maturity levels very low (0.00–0.29), low (0.30–0.49), intermediate (0.50–0.69), advanced (0.70–0.89), and high (0.90–1.00). State funding was assessed on an ordinal scale: 0 = no state funding, 0.5 = partial funding, and 1.0 = full public coverage.

### 2.2. Statistical Analyses

To explore associations between program characteristics and performance, a Spearman rank correlation test was conducted to assess the relationship between state funding and participation rates. A non-parametric Mann–Whitney U test compared follow-up coverage between HPV-based and cytology-based screening modalities. In addition, a user-friendly visualization included a color-coded heatmap to illustrate country-level PMI variability.

All analyses were performed using Python version 3.13.0 (pandas, seaborn) and R version 4.5.1, applying a significance threshold of *p* < 0.05 where statistical tests were used.

## 3. Results

### 3.1. National Guidelines, Program Organization, and Invitation Systems

**Figure 1:**
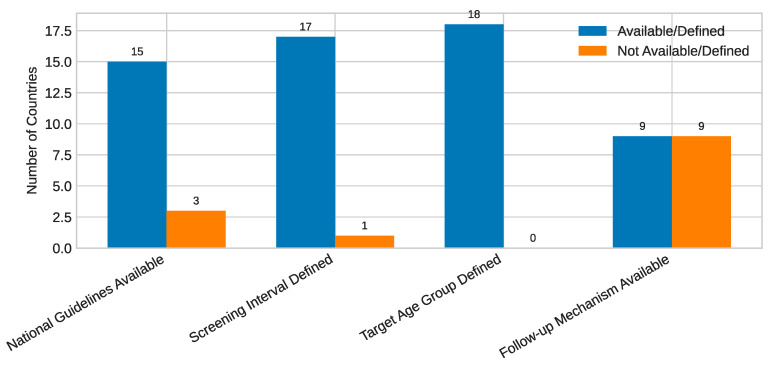
Availability of national guidelines.

Among the eighteen national and territorial submissions, fifteen reported the existence of national cervical screening guidelines, while seventeen defined a regular screening interval, most commonly three or five years (Figure 1). Overall, nine submissions identified their screening program as organized, six as opportunistic, and three as mixed.

While detailed national descriptions are provided below, the section highlights regional contrasts and trends in program organization and invitation systems.

Regarding the existence of formal cervical screening guidelines, the scope, recency, and level of implementation varied significantly across countries. In Albania, national guidelines are available and were last updated in 2019. Armenia adopted a National Guideline for Cervical Cancer Screening in 2025, coinciding with the transition to HPV-based screening pilots. Belarus has one of the most comprehensive and recently updated frameworks, with a Ministry of Health Decree (No. 174, 16 December 2024) on periodic adult health examinations and a Ministry Order (No. 389, 4 April 2025) outlining procedures for early diagnosis of precancerous and tumor diseases.

Georgia operates under a National Guideline for Cervical Cancer Screening and Diagnosis, updated in 2022. Kazakhstan reported a national guideline established in 2012, while Türkiye also referenced a 2012 guideline that underpins its five-year screening interval and discontinuation criteria. In Kosovo (UNSCR 1244), the national guideline is under revision as part of broader program development. Moldova’s guidelines were last updated in 2020. North Macedonia applies a 3-year Pap cytology program under the National Commission on Prevention and Early Detection of Cervical Cancer, which is now preparing a transition plan toward HPV-based screening.

The Kyrgyz Republic has a two-tiered system, with a 2020 clinical practice guideline for PHC-level evaluation and referral, and a 2024 clinical guideline covering advanced diagnostic and therapeutic protocols. Serbia operates under a National Program for Early Detection of Cervical Cancer developed in 2013, while Turkmenistan adopted new National Guidelines on Cervical Cancer Screening in 2024. Tajikistan and Bosnia and Herzegovina (Federation BiH, Republika Srpska, and Brčko District) remain in transition phases, with pilot protocols informing the development of national frameworks. Uzbekistan cited a Presidential Decree (#402) mandating implementation of national cervical and breast cancer screening programs.

This heterogeneity highlights an uneven policy landscape across the region. Some countries—such as Belarus, Georgia, and the Kyrgyz Republic—have well-structured and updated frameworks, whereas others are still operating under pilot-level or decentralized models.

Invitation and outreach mechanisms likewise showed substantial variation. Albania uses formal letters from primary health-care centers as part of an organized recall system; Armenia relies on outreach through PHC providers; Belarus and Türkiye use digital invitation systems integrated with national registries; and Georgia combines municipal outreach with a centralized digital booking platform. In contrast, Bosnia and Herzegovina, the Kyrgyz Republic, and Ukraine rely primarily on manual outreach by PHC workers. Moldova, Serbia, and Turkmenistan have intermediate models combining electronic and manual recall.

Quantitative invitation coverage, available from four submissions, ranged from 23% to 100%. Turkmenistan reported full invitation coverage (100%) among women aged 29–55 between 2022 and 2025, while Tajikistan reported 94.2% coverage in pilot districts. Serbia’s regional estimates ranged from 35% to 68%, and Uzbekistan reported inviting around 700,000 women annually. The lack of tracking systems in several countries limits reliable assessment of population reach.

Public financing models also vary widely. Twelve countries reported at least partial state funding, including Kazakhstan and Belarus, where screening is fully financed through public systems. Tajikistan and parts of Bosnia and Herzegovina rely more heavily on private provision, while Kosovo’s municipal programs are donor-supported but plan for national coverage.

In summary, despite broad policy adoption, operational maturity varies widely, with follow-up and digital invitation systems remaining the weakest elements.

### 3.2. Screening Intervals and Modalities

Across the 18 national submissions, 17 reported an official cervical screening interval, while only Tajikistan had no nationally defined policy and continues to rely on pilot protocols. The most common intervals were three and five years. Six countries—including Serbia, Turkmenistan, North Macedonia, Moldova, and Kosovo (UNSCR 1244)—adopted a 3-year interval, and four (Armenia, Türkiye, Ukraine, and Uzbekistan) adopted a 5-year interval. Intervals varied from annual in Federation BiH to seven-year in Albania, four-year in Kazakhstan, and mixed three- to five-year approaches in Georgia. Belarus applied age-stratified intervals, with cytology every three years for younger women and HPV testing every five to six years for older groups.

This variety reflects the absence of harmonized regional standards and underscores the importance of aligning policies with international recommendations, particularly regarding test-specific intervals.

**Figure 2:**
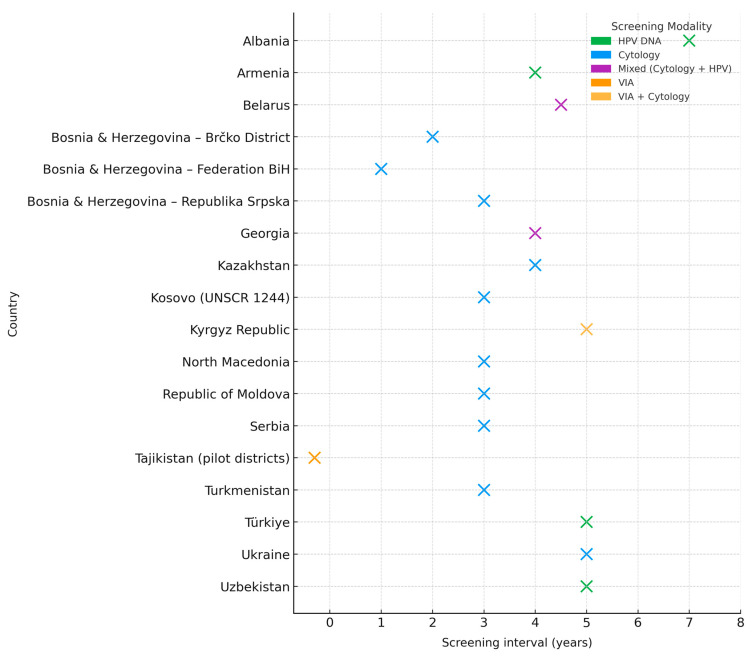
Screening intervals and modalities.

Screening methodologies also varied considerably. Pap cytology remains the most common method, used in seven countries, while six have adopted HPV DNA testing as the main approach (Figure 2). Two reported combined Pap/HPV algorithms, and the Kyrgyz Republic still uses VIA in some facilities. Several countries are in transition: Serbia and Georgia are piloting HPV-based testing; Belarus has introduced age-stratified protocols; and Uzbekistan is scaling up HPV testing through a multi-tiered delivery model.

The diversity of approaches demonstrates that program design—rather than test technology alone—determines effectiveness. Countries with registry-based systems and structured follow-up achieve higher participation and more consistent performance, regardless of screening modality.

### 3.3. Service Delivery Models

Service delivery models for cervical screening across EECA countries reveal significant differences in integration and health-system maturity. In Albania, Armenia, Belarus, Kazakhstan, Moldova, and Serbia, screening is delivered mainly through public primary-health-care networks with integrated gynecology services. Georgia applies a mixed model centered on OB/GYN specialists and municipal screening centers, while Türkiye provides services through Cancer Early Diagnosis and Screening Centers (KETEM).

**Figure 3:**
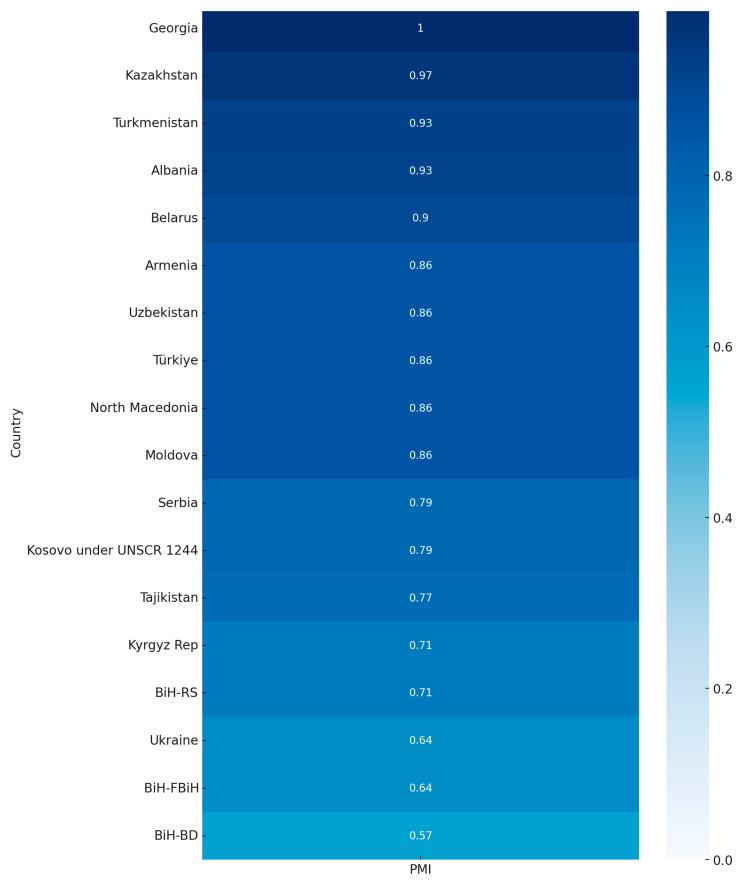
Program Maturity Index (Heatmap).

Turkmenistan and Uzbekistan operate structured, tiered delivery models linking primary, secondary, and tertiary levels of care through defined referral pathways (Figure 3). Ukraine and Bosnia and Herzegovina rely on decentralized systems where screening is mostly opportunistic and uncoordinated. The Kyrgyz Republic provides Pap and VIA screening in about 40% of PHC facilities, with tertiary referral for abnormal findings.

Countries with centralized registries and defined referral chains—such as Belarus, Turkmenistan, and Uzbekistan—report stronger linkage to diagnostic and treatment services. In contrast, fragmented systems often lack continuity between screening and care, resulting in lower effectiveness. Integration within PHC and clear referral mechanisms remain key determinants of performance and follow-up outcomes across the region.

### 3.4. Financing Models and Follow-Up Systems

Across the region, financing models for cervical screening show marked diversity. Most countries fund screening through state budgets or health-insurance schemes—such as Belarus, Georgia, Kazakhstan, Moldova, North Macedonia, Serbia, and Türkiye—while others maintain mixed models involving co-payments or donor contributions (Armenia, Tajikistan, Turkmenistan, Uzbekistan). In Bosnia and Herzegovina, financing depends on entity-level arrangements, and in Ukraine, screening often remains out-of-pocket despite policy inclusion.

Follow-up of screen-positive women emerged as the weakest and most inconsistent program component. Albania reported 90% follow-up, Belarus 100%, Moldova 89%, and Georgia 52.5% nationally (94% in Tbilisi). Kazakhstan reported 1.44%, Turkmenistan 19.8%, and Tajikistan 100% within its pilot districts. Other countries—Armenia, Kyrgyz Republic, North Macedonia, Serbia, Türkiye, Ukraine, and Uzbekistan—did not report quantitative data, indicating gaps in monitoring and referral tracking.

This variability confirms that despite wide adoption of guidelines and public financing, program effectiveness remains limited by weak follow-up mechanisms. The absence of robust patient-tracking systems delayed diagnostic confirmation, and fragmented coordination between care levels remain major obstacles to achieving equitable outcomes.

Comprehensive electronic registries and systematic patient navigation are crucial to ensure that women who screen positive are promptly linked to diagnosis and treatment, transforming policy intent into measurable public-health impact.

### 3.5. Comparative Analysis

The following comparative analysis integrates these descriptive findings to explore quantitative relationships between program maturity, state financing, and performance outcomes. Participation was reported by six submissions: four organized programs (Albania, Belarus, Kazakhstan, Turkmenistan), one opportunistic program (Tajikistan, in a pilot context), and one mixed system (Georgia). Descriptive participation rates by program type are shown in Table S1, highlighting a median of 57.2% in organized systems compared with 15.0% in opportunistic settings. A non-parametric Mann–Whitney U test comparing organized and opportunistic submissions did not reach statistical significance (U = 4.0, *p* = 0.40), as expected given the very small sample and the single opportunistic observation. Pooling non-organized systems (opportunistic + mixed; n = 2) and comparing them against organized programs (n = 4) likewise yielded a non-significant result (U = 4.0, *p* = 1.00). Despite this, the descriptive gap is clear: the organized-program median (57.2%) exceeds the opportunistic figure (15.0%) by over 40 percentage points, highlighting the practical advantage of structured, population-based models.

Quantitative invitation coverage was reported by only four submissions (Serbia, Tajikistan, Turkmenistan, and Uzbekistan). Reported figures ranged from 23% in Uzbekistan to 100% in Turkmenistan, with Serbia providing subnational estimates between 35% and 68%. The overall range among reporting countries was therefore 23–100%, with a mean of 56.1% and a median around 68%. Tajikistan provided pilot-only data and was not included in this pooled calculation. The scarcity of reporting prevented correlation analysis between invitation coverage and participation rates, except in Turkmenistan, which documented both (100% invitation coverage and 91.5% participation).

Follow-up of screen-positive women displayed the widest variability among all indicators. To enable comparison, countries were grouped into three categories: low (<20%), medium (20–80%), and high (>80%). Using this approach, Kazakhstan (1.44%) and Turkmenistan (19.8%) fell into the low group; Georgia at the national level (52.5%) represented the medium group; and Albania (90%), Moldova (89%), Belarus (100%), and the Tajikistan pilot (100%) were classified as high. While more than half of reporting countries achieved high levels of follow-up, a substantial minority remain at critically low levels. Because the distribution is bimodal—clustered at both extremes—summary measures such as the median or mean would be misleading and were therefore not reported. Instead, the coefficient of variation (CV) for follow-up was 47%, statistically confirming that this indicator is far more heterogeneous than other structural features of screening programs. These results underline that strong performance is achievable, but only under conditions where electronic registries and systematic tracking are in place.

Financing was also assessed in relation to program performance. Thirteen submissions reported full state financing of screening and follow-up, while others depended on mixed models with out-of-pocket contributions or donor funding. Among the six submissions with both financing and participation data, the Spearman rank correlation was weak and not significant (ρ = 0.21, *p* = 0.69). This indicates that while public financing is a prerequisite for equity, program performance depends primarily on organizational features such as electronic registries, systematic invitations, and tracked follow-up.

Finally, the association between screening modality and participation was explored. Among organized programs that reported participation, HPV-primary approaches (Albania 40%, Belarus 19% within a stratified algorithm) did not outperform cytology-based or combined approaches (Kazakhstan 74.4%, Turkmenistan 91.5%). With so few observations, differences cannot be attributed to test type. The results suggest that program organization and system capacity, rather than screening modality, drive participation and follow-up outcomes.

### 3.6. Program Maturity Index (PMI)

The Program Maturity Index (PMI) was developed to summarize the structural and performance maturity of cervical screening programs in the Eastern Europe and Central Asia (EECA) region. Eight indicators were selected based on WHO and European quality assurance frameworks. Each indicator was scored on a binary or scaled basis, and the mean of all scores was used to derive a composite PMI value for each national submission. Equal weighting was applied across indicators due to the exploratory nature of the index. The PMI is intended as an illustrative benchmarking tool to visualize regional variability and identify areas for system strengthening, rather than a definitive ranking of program performance (Figure 3). A detailed description of scoring, weighting, and indicator definitions is provided in Supplementary Table S2. The PMI is exploratory and was not designed for inferential comparison.

The PMI analysis demonstrated that the region overall is at an intermediate to advanced stage of program development. The regional mean PMI was 0.73 (median 0.83; range 0.00–1.00), indicating that, on average, countries in EECA have implemented nearly three-quarters of the essential building blocks of a comprehensive cervical screening program. Using interpretation thresholds, ten countries were classified as advanced (0.70–0.89), five as high (≥0.90) and three as intermediate (0.50–0.69). These results highlight wide disparities across the region: while several systems have reached a high level of maturity, others remain at the very early stages of program development.

The strongest contributors to higher PMI values were the presence of national guidelines (15/18 submissions), definition of target groups (18/18), and organized program structures (9/18). By contrast, the weakest component was functional follow-up of screen-positive women, which was reported quantitatively by only seven countries and varied dramatically from below 2% to over 90%. This gap substantially constrained maturity scores even among otherwise well-structured programs.

While Georgia’s cervical screening program is formally classified as a mixed model—combining opportunistic and organized elements—it achieved the highest Program Maturity Index (PMI) score in the region (0.996). This result reflects not only the availability of core policy components, but also Georgia’s exceptional investment in the operational aspects of program delivery. In particular, the country has developed a robust digital infrastructure, including a centralized electronic registry and a digital invitation system that supports individual tracking and recall. Although the program combines both Pap cytology and HPV testing, its performance in areas such as data management, municipal outreach, and follow-up coordination has enabled pilot regions to achieve participation rates as high as 97%. These operational strengths suggest that the PMI is sensitive to real-world system functionality—such as registry-enabled monitoring and patient navigation—rather than relying solely on formal program classification. Georgia’s case illustrates how a hybrid model, if well-managed and digitally enabled, can outperform nominally organized systems that lack integration or follow-up capacity.

Although equal weighting was applied across all eight indicators in the PMI calculation, qualitative interpretation placed greater emphasis on those related to continuity of care, specifically structured follow-up and linkage to treatment after positive screening. This interpretive emphasis does not modify the quantitative weighting but aligns with established quality-assurance frameworks, including the WHO Global Strategy and European Guidelines for Quality Assurance in Cervical Cancer Screening [6,11]. These frameworks consistently identify incomplete follow-up as the main determinant of program effectiveness. Similar multidimensional indices in health-system performance monitoring have combined equal mathematical weighting with interpretive prioritization of key outcome domains to preserve transparency while reflecting public-health relevance [5,7].

The PMI therefore provides a useful summary measure to illustrate inter-country variability and highlight priority areas for investment. Nevertheless, given data gaps and the reliance on self-reported information, it should be interpreted as exploratory and illustrative rather than as a definitive benchmarking tool. Its main value lies in identifying structural strengths, such as widespread adoption of national guidelines, and persistent weaknesses, such as limited follow-up, that must be addressed if the region is to reach the WHO 2030 cervical cancer elimination targets.

## 4. Discussion

This study provides the most comprehensive comparative assessment to date of cervical screening systems across 18 submissions from 16 EECA countries and territories. By combining structural, policy, and performance indicators, it offers a clear picture of a region in transition: while many countries have made notable progress in aligning with international recommendations, persistent weaknesses in program organization and follow-up continue to undermine impact.

The findings confirm international evidence that organized, population-based programs consistently outperform opportunistic models. Median participation in organized systems exceeded opportunistic approaches by over 40 percentage points, despite similar levels of financing. This underscores that the essential determinant of program effectiveness is not the presence of guidelines or funding alone, but the establishment of systematic population registries, electronic invitation systems, and active recall mechanisms. Opportunistic programs, even when technically supported and partially financed, remain unable to achieve population coverage or equity.

Encouragingly, fifteen of eighteen submissions reported the existence of national screening guidelines and seventeen reported defined screening intervals, reflecting political recognition of cervical cancer prevention as a public health priority. However, the presence of guidelines has not always translated into effective implementation. Bosnia and Herzegovina illustrates the risks of health system fragmentation: three administrative parts (Federation BiH, Republika Srpska, and Brčko District) under different or absent protocols, with intervals ranging from annual to triennial and no unified policy. Similar gaps are visible in Tajikistan, where the absence of national guidelines leaves pilot projects to function in isolation. These cases highlight that formal policy adoption is a necessary but insufficient condition: only when accompanied by adequate financing, monitoring systems, and institutional accountability can guidelines lead to improved outcomes.

The most critical weakness emerging across the region is the absence of structured follow-up systems for screen-positive women. Although nearly all submissions reported that diagnostic services such as colposcopy and biopsy are available in principle, only seven countries provided quantitative data on follow-up coverage. Even within organized programs, reported performance was often very low, in some cases close to one percent. This represents a systemic threat to the effectiveness of screening: without reliable linkage from a positive screening test to diagnostic confirmation and timely treatment, early detection cannot achieve its intended impact on incidence and mortality. The gap is especially concerning in light of the WHO Global Strategy for the Elimination of Cervical Cancer, which explicitly targets treatment for 90% of women identified with cervical precancer or cancer. Closing this gap will require coordinated policy action, including investment in electronic patient-tracking systems, the establishment of clear referral protocols across levels of care, and the integration of performance monitoring and incentives for health facilities to ensure that women who screen positive are not lost to follow-up.

Financing analysis showed no significant correlation with participation rates, largely because most programs already reported full state coverage. Yet inequities remain. In several settings, including Federation BiH, Brčko District, and Tajikistan, women continue to rely on private payment for screening. This threatens universal access and risks reinforcing socioeconomic inequalities in health outcomes. Policy frameworks should therefore prioritize universal public coverage of both screening and follow-up, while linking financing to performance indicators to ensure that resources translate into effective service delivery.

Contrary to expectations, HPV-based programs did not outperform cytology-based systems in participation. Albania and Belarus reported relatively low uptake despite adopting HPV as the primary test, while cytology-based programs such as Kazakhstan and Turkmenistan reported higher participation. This suggests that technological choices cannot compensate for weak system design. Policymakers should thus avoid framing cervical cancer elimination as a purely technological challenge. Rather, investments in HPV testing should be embedded within organized, population-based frameworks that ensure invitations, recalls, and robust follow-up. Similar findings have been reported in Thailand, where HPV self-sampling increased screening uptake within organized programs, suggesting that sampling strategy rather than test type is the main driver of participation [13].

From a regional perspective, these findings emphasize the urgent need for coordinated policy action. Countries with mature programs—such as Belarus, and advanced programs in Turkmenistan and Kazakhstan, though follow-up remains limited—can serve as reference models, sharing protocols, electronic tools, and training resources with those in earlier stages. Regional alliances, including the EECA Cervical Cancer Prevention Network, should facilitate peer learning, promote harmonization of quality standards, and advocate for integration of screening with HPV vaccination strategies. Bosnia and Herzegovina is example of existence of different health systems (decentralized in the Federation of Bosnia and Herzegovina and centralized in the Republika Srpska and Brčko District) providing opportunistic services. It is important to note that in all parts of BiH, comprehensive research on multiple cancer types is ongoing to inform the development of future organized cervical screening programs; by contrast, countries like Georgia and Uzbekistan demonstrate how new guidelines and multi-tiered models can create momentum for system reform.

Weak follow-up directly undermines progress toward WHO’s 90–70–90 targets, particularly the goal of 90% treatment coverage for screen-positive women, and its clinical implications are profound. In regions where population coverage is low and tracking systems are incomplete, many women are diagnosed at advanced or even metastatic stages of cervical cancer. In such cases, survival depends on multimodal strategies that combine systemic therapy with individualized local treatment. Case-based evidence [14] has shown that even in metastatic cervical adenocarcinoma, carefully selected multimodal approaches can improve prognosis and provide meaningful survival benefits in what is usually considered a non-curable setting. Likewise, the pivotal trial by Tewari et al. [15] demonstrated that adding bevacizumab to chemotherapy improved overall survival in patients with recurrent, persistent, or metastatic cervical cancer. These examples underline that strong screening and follow-up systems are not only preventive tools but also crucial to reducing the clinical and financial burden of late-stage, resource-intensive management. Emerging evidence also emphasizes that screening alone is not sufficient; integrating HPV vaccination with risk-stratified approaches and genetic biomarkers can further advance precision prevention [16]. Policymakers should therefore view screening not as a standalone intervention but as a continuum of care. Establishing performance-linked financing, investing in digital registries, and embedding monitoring and evaluation into national cancer control strategies are critical policy actions to ensure that EECA countries move decisively toward elimination

## 5. Policy Recommendations

Several policy priorities emerge from this study for accelerating cervical cancer elimination in Eastern Europe and Central Asia.

Countries should prioritize the transition from opportunistic to organized, population-based screening. Establishing population registries, electronic invitation and recall systems, and standardized follow-up protocols is essential to achieving high coverage and equitable access. Robust follow-up systems must become a central policy focus. The effectiveness of screening depends on ensuring that women with positive results are linked to timely diagnosis and treatment. Investment in digital patient tracking, clear referral pathways, and accountability mechanisms should therefore be core elements of national cancer control strategies. Financing should be universal, sustainable, and performance-linked. Public coverage of screening, diagnosis, and treatment is necessary to secure equity, but linking financing to participation and follow-up indicators will ensure that resources are translated into measurable outcomes. Screening policies should also be harmonized with HPV vaccination strategies as part of an integrated approach to cervical cancer elimination. Vaccination scale-up provides opportunities to build shared registries, strengthen communication strategies, and enhance the credibility of prevention programs. Finally, regional collaboration should be leveraged as a catalyst for progress. The Cervical Cancer Elimination Strategy [12] provides an overarching global policy framework that has already mobilized political attention and technical resources. Within the EECA region, the UNFPA EECA Alliance for Cervical Cancer Prevention plays a strategic role in uniting ministries of health, civil society, and technical partners [2]. The Alliance can serve as a regional platform for peer learning, sharing of digital tools and clinical protocols, coordination of training, and collective advocacy for harmonized policies aligned with WHO’s global targets. By aligning national commitments with this global strategy and leveraging the coordinating role of the regional Alliance, EECA countries can accelerate progress toward the WHO “90–70–90” milestones and position the region as an early achiever in cervical cancer elimination, thereby reducing the number of women presenting with advanced, resource-intensive disease.

## 6. Conclusions

This study provides the most updated comparative overview of cervical screening programs across Eastern Europe and Central Asia. While several countries have adopted national guidelines and achieved moderate-to-advanced program maturity, persistent weaknesses in follow-up systems and data infrastructure remain major barriers to achieving the WHO 90–70–90 elimination targets. Strengthening national electronic registries, ensuring universal public financing, and enhancing regional collaboration through the catalytic role of the UNFPA Regional Alliance for Cervical Cancer Prevention are essential to accelerate progress. The findings underscore that program organization and system capacity, rather than screening modality alone, determine real-world effectiveness and equity in cervical cancer prevention.

## 7. Study Limitations and Strengths

This study is cross-sectional and based on self-reported data. Despite internal validation, responses may reflect interpretation differences or incomplete monitoring, introducing potential reporting bias. While this ensured comparability across submissions, it also introduced the risk of reporting bias, particularly where indicators were incomplete or subject to interpretation by national respondents. Second, quantitative data on participation, invitation coverage, and follow-up were only available from a subset of countries. This restricted the scope of statistical analysis and limited the ability to test associations with sufficient power. For example, the observed differences in participation between organized and opportunistic programs were large but could not be confirmed statistically due to the very small number of opportunistic data points. Third, data completeness varied across indicators. Several countries reported the availability of services (such as diagnostic follow-up) without providing quantitative coverage, which makes it difficult to distinguish between genuine capacity gaps and incomplete monitoring systems. Similarly, in some cases, reported high follow-up rates likely reflected pilot or municipal initiatives rather than national program performance. Fourth, the Program Maturity Index was developed as an illustrative tool to synthesize heterogeneous indicators into a single metric. While it offers a useful overview of inter-country variability, it cannot fully capture the complexity of program performance and should not be interpreted as a definitive ranking. The PMI’s validity is constrained by the uneven availability of data and the need to apply simplifying assumptions (e.g., coding financing into ordinal categories). Finally, the study provides a cross-sectional snapshot as of 2024–2025. Several countries are undergoing reforms, piloting HPV-based screening, or revising national guidelines. These transitional dynamics mean that the findings may not fully reflect rapid policy shifts expected in the coming years.

Despite these limitations, the study has several important strengths. It is the first to compile and systematically analyze national-level data on cervical screening from 18 submissions across 16 EECA countries and territories, using a standardized tool aligned with WHO and European guidance. By combining policy, structural, and performance indicators, it provides a uniquely comprehensive overview of program implementation in a region where evidence has previously been sparse. The inclusion of diverse systems—from fully organized to opportunistic—allowed for meaningful comparisons that highlight the central role of program organization in determining outcomes. The development of the Program Maturity Index, while illustrative, also offered a useful means of benchmarking countries against international standards and identifying common gaps such as follow-up. Finally, the integration of these findings with regional policy frameworks, including the UNFPA Cervical Cancer Elimination Strategy [12] and the UNFPA EECA Alliance for Cervical Cancer Prevention [2], ensures that the study not only describes the current landscape but also provides directly actionable insights for policymakers.

## Data Availability

The data presented in this study were derived from the following resources available in the public domain: National Ministries of Health, WHO at https://www.who.int/data/gho/indicator-metadata-registry/imr-details/3240 (accessed on 20 August 2025), IARC databases at https://gco.iarc.fr/en (accessed on 20 August 2025) and https://screening.iarc.fr/cervicalimagebank.php (accessed on 20 August 2025) and UNFPA at https://eeca.unfpa.org/en/regional-alliance-cervical-cancer-prevention (accessed on 20 August 2025).

## Supplementary Materials

The following supporting information can be downloaded at: https://www.mdpi.com/article/10.3390/healthcare13222889/s1, Table S1: Description of national program classification and indicators used for the Program Maturity Index (PMI).; Table S2: Detailed scoring matrix and country-specific PMI results.
