# Supplementary material for: Cervical Screening Systems in Eastern Europe and Central Asia: A Comparative Policy Evaluation
Source: Healthcare (Basel). 2025 Nov 13;13(22):2889. doi: 10.3390/healthcare13222889 (PMC12652621; doi:10.3390/healthcare13222889)
Supplement: Supplementary file 1 [file healthcare-13-02889-s001.zip › Table_S2_Detailed scoring matrix and country-specific PMI results.pdf]

Table S2. Detailed scoring matrix and country-specific PMI results

Indicators, Scoring, and Weighting Used in the Program Maturity Index (PMI)

Indicator	Description	Scoring Scale	Weight	Data Source / Notes
Program type	Existence of an organized, population-based screening program with defined invitation and recall mechanisms	Organized = 1; Opportunistic = 0	1.0	National program description
National guideline available	Presence of officially adopted national guideline or protocol for cervical cancer screening	Yes = 1; No = 0	1.0	Ministry of Health documents / national reports
Defined screening interval	Existence of officially specified screening interval in national policy	Yes = 1; No = 0	1.0	National reports
Defined target age group	Existence of officially defined target age group for screening	Yes = 1; No = 0	1.0	National reports

Invitation system	Existence and type of population invitation or recall system	Centralized/digital = 1; Partial/manual = 0.5; None = 0	1.0	Program description
Follow-up mechanism	Availability and quantitative coverage of follow-up for screen-positive women	≥80% = 1; 20–79% = 0.5; <20% = 0	1.0	National monitoring data
Financing coverage	Extent of public/state financing for screening and follow-up	Full = 1; Partial = 0.5; None = 0	1.0	National health financing data
Participation rate	Proportion of invited women who underwent screening during reporting period	≥70% = 1; 30–69% = 0.5; <30% = 0	1.0	Reported program data

Interpretation thresholds

Category	PMI Range	Interpretation
Very low	0.00–0.29	Early or pilot stage; limited program structure
Low	0.30–0.49	Initial policy framework; weak implementation
Intermediate	0.50–0.69	Partial implementation; developing systems
Advanced	0.70–0.89	Established systems with good coverage
High	0.90–1.00	Fully implemented and mature system

The PMI for each submission was calculated as the arithmetic mean of all eight indicator scores:

PMI = $(\Sigma x_i) / 8$, where x_1–x_8 represent standardized indicator values.
